# Processing and Characterization of Polymer-Based Far-Infrared Composite Materials

**DOI:** 10.3390/polym11091451

**Published:** 2019-09-04

**Authors:** Yabo Xiong, Yang Zou, Shaoyong Cai, Huihui Liu, Shaoyun Huang, Houbin Li

**Affiliations:** 1 School of Printing and Packaging, Wuhan University, Wuhan 430079, China; 2 Institute of Chemical Materials, China Academy of Engineering Physics, Mianyang 621999, China

**Keywords:** ceramic powders, far-infrared emissivity, composites, polymer

## Abstract

Polymer-based far-infrared radiation (FIR) composite materials are receiving increasing attention due to their significant influence on bioactivity. This study reports the processing of FIR composite films based on a polymer matrix and FIR radiation ceramic powders, as well as the characterization of the FIR composites. Field-emission scanning electron microscopy (SEM) and laser particle size analysis were employed to analyze the characteristic of the ceramic powders. The average size, dispersity, and specific surface area of the ceramic powders were 2602 nm, 0.97961, and 0.76 m^2^/g, respectively. The results show that the FIR ceramic powders used in the composite films had excellent far-infrared emissive performance. Moreover, by using differential scanning calorimetry (DSC) and thermogravimetric analysis (TG), it was indicated that the thermal performance and mechanical properties of the composite films were significantly influenced (*p* < 0.05) by the addition of the FIR ceramic powders. Specifically, the elongation at break decreased from 333 mm to 201 mm with the increase in FIR ceramic powders. Meanwhile, the contact angle and light transmittance were also changed by the addition of the FIR ceramic powders. Furthermore, the two different processing methods had great influence on the properties of the composite films. Moreover, the composite blown films with 1.5% FIR ceramic powders showed the highest far-infrared emissivity, which was 0.924.

## 1. Introduction

Previous studies reported that far-infrared radiation (FIR) is an invisible form of electromagnetic solar energy which has significant biological effects, such as improving blood flow, influencing cell fractions in the blood and cytokine production, and activating the self-defense functions of the body. Furthermore, FIR radiation can penetrate biological tissue and have a strong rotational and vibrational energy effect at the molecular level; therefore, it was applied to explore its cellular mechanism in promoting neurite outgrowth and possible neural regeneration [1,2]. Furthermore, FIR has the ability to activate water molecules, which is responsible for the diverse biological effects [3,4]. In addition, researchers also found that the underlying biophysical mechanisms of the interaction of electromagnetic radiation with living cells can be framed in terms of altered cell membrane potentials and altered mitochondrial metabolism [5]. Since the absorption of FIR can cause resonance within biological cells, far-infrared radiation can transfer energy to organisms, resulting in a wide variety of biological effects. Moreover, it was reported that boron–silicate mineral, tourmaline, and bamboo charcoal, which can emit far-infrared rays, were incorporated into fibers to make FIR composites [6,7,8,9]. Furthermore, ceramic powders were studied on account of their far-infrared emissivity properties [10,11,12]. Given the FIR emissivity properties of ceramic powders, they could become promising packaging fillers for improving the functionality of packaging materials.

Due to their excellent mechanical and thermal properties, as well as good electrochemical performance, polymers play an important role in the preparation of composites [13,14]. In particular, plastic materials, such as nylon, polythene, and polypropylene, are widely used in a variety of applications as a result of their excellent properties including low density, high specific strength, low cost, and ease of processing. In order to further improve these properties, different functional fillers could be added into the polymer. Composite technology is applied widely; for example, a novel artificial material was fabricated by combining ceramic and polymer materials in an ordered manner or by simple mixing. However, in previous research, large numbers of ceramic–polymer composites were introduced for telecommunication and microelectronic applications [15]. In addition, polymer-based FIR composites were proposed for other applications, such as textile fabrics, optical applications, coatings, and nano-membranes. Because they can emit the energy absorbed from the environment as far-infrared rays, the novel composites attracted the attention of researchers [16,17,18].

Therefore, the combination of far-infrared radiation ceramic powders and polymer materials is a promising way to improve the properties of composite materials. In this work, four different proportions of ceramic powders were added into low-density polyethylene. Eight different types of composite films were fabricated by using a film-blowing machine and a hot-pressing machine. Subsequently, the characteristics of the composite films were investigated.

## 2. Materials and Methods

### 2.1. The Characterization of Ceramic Powders

The FIR ceramic powders were provided by Zhuhai Deshen Environment-Friendly Packaging Co., Ltd., China. The polymer pellets of low-density polyethylene were purchased at Sinopec. A scanning electron microscope (SEM) and laser particle size analyzer were employed to observe the surface features, shape, and size of the sampled particles of the FIR ceramic powders. The FIR ceramic powders were coated with gold 30 min prior to observation by SEM, then observed at magnifications of 500× and 5000×. The measuring conditions of the laser particle size analyzer were as follows: laser wavelength = 365 nm, scattering angle = 90°, and measuring time = 180 s; water was used as the medium at room temperature. X-ray fluorescence spectrometry (XRF, Bruker AXS, Karlsruhe, Germany) and an X-ray diffractometer (XRD-7000, Shimadzu, Kyoto, Japan) were used to determine the inorganic compositions of the FIR ceramic powders as reported in a previous article. XRF was used under a spectrum system (element range: ^4^Be–^92^U, resolution: 129 eV, electrically cooled). XRD scans were obtained using Cu-Kα radiation over a scanning range from 5° to 40°.

### 2.2. The Processing of Composite Films

**Figure 1:**
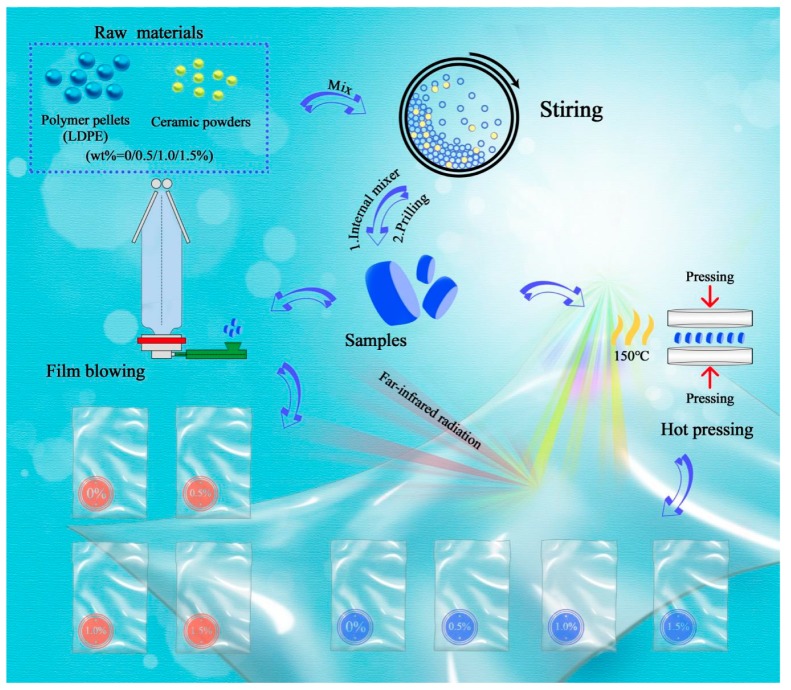
Flow chat of method for making eight different types of composite films.

Virgin polymer pellets of low-density polyethylene (LDPE) blended with FIR ceramic powders in four ratios (*w*/*w*; 0.0%, 0.5%, 1.0%, 1.5%) were fed into an internal mixer for 300 s at 150 °C; then, the pelleting process was performed using a single-screw extruder under controlled rotational speed. Eventually, the blended polyester pellets were processed into composite films using the film-blowing machine and hot-pressing machine. As demonstrated in Figure 1, the polyester pellets with four different concentrations of FIR ceramic powders were manufactured into eight types of films using two different processing methods. The first four films (1–4) contained 0.0%, 0.5%, 1.0%, and 1.5% FIR ceramic powder, formed by hot-pressing at 150 °C, while the remaining films (5–8) were generated by film-blowing, and contained 0.0%, 0.5%, 1.0%, and 1.5% FIR ceramic powders. When testing the mechanical properties, the direction of composite film forming was set as the *y*-axis, and the *x*-axis was the corresponding orthogonal direction. The FIR ceramic powders were evenly dispersed in the films. The control groups without FIR ceramic powders (films 1 and 5) were prepared for comparison. After cooling at room temperature, the composite films were cut to the same size (25 cm × 25 cm).

### 2.3. Characterization of Composite Films

Thermal analysis was performed using differential scanning calorimetry (DSC, NETZSCH, Selb, Germany) and thermogravimetric analysis (TG 209, NETZSCH, Selb, Germany). The investigated temperature range of DSC was between 25 °C and 200 °C. In order to eliminate the influence of thermal treatment, the samples were heated at a rate of 40 °C/min from 25 °C to 200 °C, held at 200 °C for 5 min, cooled at a rate of 10 °C/min to 25 °C, held for 5 min, then heated again at a rate of 10 °C/min from 25 °C to 200 °C. Each sample was weighed and sealed in an aluminum vessel. Thermogravimetric analysis was carried out by a TG analyzer, where 8–10 mg of sample was placed in a platinum crucible and heated from 25 °C to 600 °C at a rate of 15 °C/min under nitrogen flow (30 mL/min). The mechanical properties in the longitudinal and transverse directions were characterized with a tension tester (Model 3365, Instron, Norwood, USA) by using rectangle-shaped specimens of 150 mm length and 25 mm width. The specimens were tested with a grip distance of 30 mm and a crosshead speed of 30 mm/min under ambient conditions (at 25 °C and at a humidity of 75%) using a standard method. Five specimens were analyzed for each sample, and then their values were averaged; all films were tested under the same conditions. An infrared radiometer (EMS302M, Hede, Taibei, Taiwan) was applied to analyze the FIR emissivity of the composite films. The far-infrared emissivity of the composite films was determined in the wavelength range of 5–14 μm at 25 °C using an infrared radiometer. The emissivity was measured as a relative value based on the assumption that the emissivity of a black body is 1. A black body with *ε* = 1 can convert almost all heat energy to electromagnetic energy. The contact angle measurements were carried out using a drop shape analyzer (model SL200B, Kino Industry Co., Ltd., Boston, USA), using water as a liquid of known surface tension in air. The light transmittance was calculated using a spectrophotometer (Shimadzu, A112952, Kyoto, Japan). The moisture permeability of composite films at 23 °C was determined gravimetrically according to ASTM E96M. The high-resolution morphology images of the cross-section were measured by a field-emission scanning electron microscope. The films were cryo-fractured in liquid nitrogen to obtain brittle fracture specimens. The samples of the cross-section were attached to double-sided adhesive tape and mounted on the specimen holder, before being sputtered and coated with gold under vacuum before observing.

### 2.4. Statistical Analysis

The samples were studied using a randomized design. The experimental data are presented as means ± standard deviation (SD) for three replicate samples. Significant differences between films were determined using Duncan’s multiple range test (*p* < 0.05). Statistical analysis was performed using SPSS 13.0 (SPSS, USA) and Origin 9.0.

## 3. Results and Discussion

### 3.1. Mineralogical Analysis of Ceramic Powders

**Figure 2:**
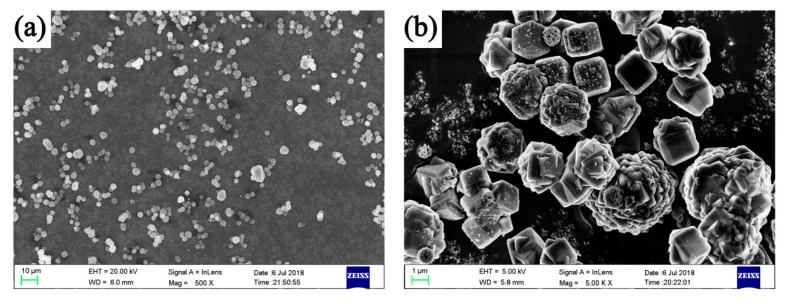
SEM images of the far-infrared radiation (FIR) ceramic powders at different magnifications.

Figure 2 shows the SEM images of the FIR ceramic powders at different magnifications. Detailed analysis based on the SEM images with the laser particle size analyzer indicated that the average size, dispersity, and specific surface area were 2602 nm, 0.97961, and 0.76m^2^/g, respectively. It is well documented that the particle size has a significant influence on the spectral intensities of the infrared range [19,20]. In correlation with the density, the presence of porosity was unnoticeable in the FIR ceramic powders. Several studies revealed that the infrared optical reflectivity measurements and numerical analysis can be used to obtain reliable information on whether the ceramic has a cubic or tetragonal unit cell. Since the emittance is dependent on its crystal structure and mineralogical composition [21,22], we investigated the crystal structure and the chemical composition of the powders. The mineralogical analysis of the FIR ceramic powders by XRF presented in our previous article showed that the FIR ceramic powders consisted of five major elements (O, Na, Al, K, and Si) and seven minor elements (Ca, S, Cl, P, Ti, Fe, and Ba) [23]; these elements had high far-infrared radioactivity [24]. As can be seen in Figure 2a,b, the FIR ceramic powders could disperse uniformly without obvious agglomeration. Therefore, they were easily added into the polymer matrix to prepare the polymer-based composite film with uniform morphology. Since the uniform morphology of he composite film is a prerequisite for far-infrared emission performance, the FIR ceramic powders were shown to emit a well-proportioned distribution of far-infrared rays, which guaranteed the accuracy of emissivity of the films [25,26]. It can be concluded that the FIR ceramic powders used in the composite film had excellent far-infrared emissive performance.

### 3.2. Mechanical Properties of Composite Films

Table 1 shows an overview of the mechanical properties of the composite films in the *x-* and *y*-directions, including Young’s modulus, length at break, tensile stress, strength at break, and energy at break. Young’s modulus is a basic mechanical parameter that characterizes the elastic deformation property of a film. A composite with a large Young’s modulus is less prone to deformation, which means it has better elasticity [27]. The significant difference (*p* < 0.05) between the two directions and two processing methods can be noted based on these five parameters in Table 1.

**Figure 3:**
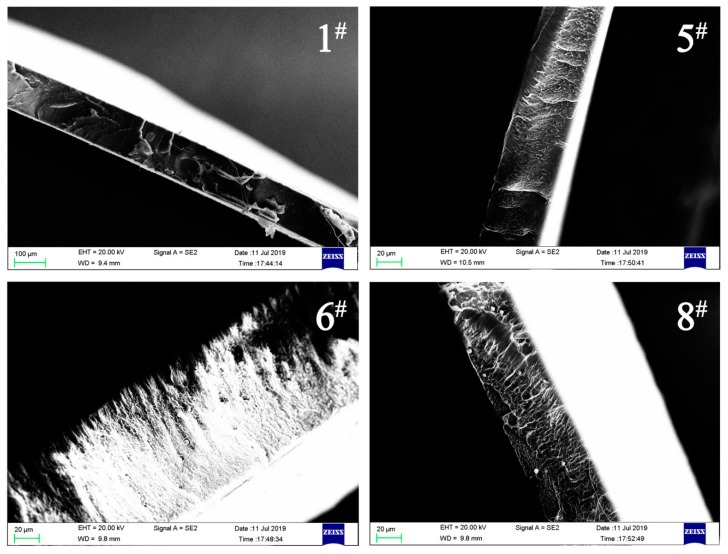
SEM images of the composite films **1**, **5**, **6**, and **8** at different magnifications.

According to Duncan’s variance analysis, the mechanical properties of the films had a significant difference between both directions. The Young’s modulus, strength at break, and energy at break along the *y*-axis were totally different from that along the *x*-axis. For example, the corresponding data of sample 1 in the *x*-axis and *y*-axis were 32.74 MPa, 56.20 N/tex, and 11.31 J, and 30.71 MPa, 9.85 N/tex, and 47.71 J, respectively. Similarly, other parameters were also different between these two directions. It can be seen in Table 1 that the differences in tensile stress and elongation at break were relatively small between both directions, while differences occurred among different composite materials. The results indicate that the mechanical properties in the *x*-axis were better than those in the *y*-axis. Meanwhile, comparing with the control samples, the tensile stress and elongation of films decreased with the increase in FIR ceramic powders in the composite films. As a result, this phenomenon may be due to the stress concentration between the FIR ceramic powders and the polymer matrix, which can cause a sharp drop in the mechanical performance of the composite film [28]. As shown in Figure 3, film 6 contained 0.5% FIR ceramic powder, while film 8 contained 1.5% ceramic powder; as the amount of FIR ceramic powder increased, the mechanical properties decreased correspondingly.

Comparing the eight types of films, the elastic deformation of the films prepared by hot-pressing was nearly 20 times larger than that of the films prepared by film-blowing along the *x*-axis, but the values were similar between films along the *y*-axis. These results indicate that the processing method of the composite films had great influence on the mechanical properties. The most striking phenomenon was that most of the mechanical parameters of the films made by blowing were nearly equivalent to those of the control films (*p* ≥ 0.05). These results indicate that the mechanical properties of the composites prepared by blowing were not significantly influenced (*p* ≥ 0.05) by the addition of the ceramic powders. However, for the composite films prepared by hot-pressing, the mechanical properties changed greatly due to the different amounts of FIR ceramic powder. Furthermore, the films prepared by hot-pressing had good mechanical ability in both directions.

### 3.3. TG and DSC Properties of Composite Films

In order to study the thermal properties of different composite films, TG and DSC were utilized. Table 2 shows the temperature scanning graph of DSC. Integral calculations indicated that the crystallinity and glass temperature of the films with the addition of FIR ceramic powders were lower than those of the films without FIR ceramic powders. As the FIR ceramic powder content increased, these values decreased gradually. This proves that the addition of FIR ceramic powders hindered the regular arrangement of polymer chain segments and led to imperfect crystallization, thereby reducing melting point and crystallinity [29].

It can be seen in Table 2 that, with the addition of FIR ceramic powders, the onset temperature, inflection temperature, and end temperature of the films with FIR ceramic powders were slightly lower than the films without FIR ceramic powders. At the same time, the mass reduction of all eight films ended at nearly 490 °C, as measured by the TG test. The residual masses of the films were 0.9%, 0.91%, 1.11%, 1.37%, 0.37%, 0.79%, 1.24%, and 1.34% in samples 1–8, respectively. This reveals that the residual mass increased with the increase in FIR ceramic powder. The method of preparation and the content of FIR ceramic powder had little effect on the onset temperature, inflection temperature, and end temperature. This may have been due to different preparation methods leading to different morphologies; these results are also consistent with the SEM.

### 3.4. Contact Angle Properties of Composite Films

The water contact angle is the spreading angle of a water droplet on the surface of an object. It is a concrete measure of the wettability, and the value directly reflects the strength of the surface wettability to water. It is also the result of a mechanical equilibrium between the interfacial tensions for different phase pairings, as formalized in Young’s equation [30], shown below.

cos θ = (γ_SV_ − γ_SL_)/γ_LV_, (1)

where γ_SV_, γ_SL_, and γ_LV_ are the interfacial tensions of solid–vapor, solid–liquid, and liquid–vapor, respectively. Contact angle is not necessarily related to the hydrophilicity of the membrane material. If the value of the water contact angle is small, it shows that the surface of the film is hydrophilic; wettability is not an intrinsic property of the material, but a surface property of the object [31].

**Figure 4:**
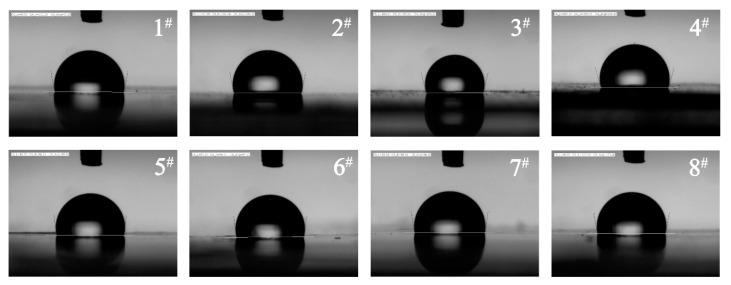
Water contact angle of eight composite films.

The analysis of contact angle was employed to investigate the surface characteristics of composite films. As can be seen in Figure 4, for the composite films formed by hot-pressing, the contact angles were 99.89°, 101.22°, 103.91°, and 106.42° in samples 1–4, respectively. The contact angles in samples 5–8 were 95.91°, 97.10°, 98.28°, and 100.06°, respectively. These results reveal that, with the increase in FIR ceramic powder content, the contact angle of the films gradually increased. They also reveal that the roughness developed by ceramic and LDPE blending can effectively increase the hydrophobicity of composites. Meanwhile, the processing methods also affected the hydrophilicity of the composites. The contact angles of control samples (1 and 5) were 99.21° and 95.91°, respectively. In total, the changes in contact angle of the films were due to the addition of the FIR ceramic powers and different processing methods.

### 3.5. Far-Infrared Emissivity, Light Transmittance, Moisture Permeability Properties, and Photomicrographs

The values of the light transmittance, moisture permeability, and far-infrared emissivity (*ε*) of the films are listed in Table 3. The light transmittance and moisture permeability properties are the basic properties of films. It can be seen that the different preparation methods had great influence on the properties of the composite films. The light transmittance of the composite films formed by blowing were five times that of the composite films formed by hot-pressing, while the moisture permeability values were only one-eighth. At the same time, it can also be seen that the addition of FIR ceramic powders increased the moisture permeability of the composite films. In order to study the dispersion behavior of FIR ceramic powders in different films, SEM was utilized. A well-proportioned dispersion can be seen in the different films.

By adding FIR ceramic powders, the emissivity of the polymers increased from 0.5 (control group) to 0.8, which is beyond the emissivity limitation of commercial products currently available [32]. Table 3 shows that the far-infrared emissivity reached about 0.92 when the FIR ceramic powder mass ratio was 1.5%. Therefore, the addition of FIR ceramic powders improved the far-infrared emissivity of the composite films. The FIR composite films have the ability to efficiently convert energy from sunlight or other sources in the environment to far-infrared radiation. The generated radiation can then be re-emitted to the surroundings, which is similar to other far-infrared emitting materials.

## 4. Conclusions

The aim of the current study was to characterize FIR ceramic powders and evaluate the performance of the designed composite films. In this work, different proportions of far-infrared radiation ceramic powders were added into low-density polyethylene, and different composite films were fabricated using a film-blowing machine and hot-pressing machine. This study focused on the effect of ceramic powders on the thermal performance, mechanical properties, and barrier properties. Lastly, the far-infrared emissivity of the composites was analyzed to evaluate their suitability for application. It was confirmed that they had the capability to emit far-infrared radiation. The most significant finding was that some mechanical properties of the composite films were significantly influenced (*p* < 0.05) by the addition of the FIR ceramic powders, which was also attributed to the film processing methods. The addition of FIR ceramic powders decreased the light transmittance, but increased the moisture permeability performance of the composite films.

This study strengthens the idea that far-infrared radiation composite films are novel and promising materials. The stimulation of water molecules or metabolism activation could be responsible for the observed positive effect. Further research needs to be undertaken to explore potential applications and the working mechanism of these films.

## Figures and Tables

**Table 1:** Mechanical properties of the composite films along the *x*-axis and *y*-axis.

	Elongation at Break (mm)	Young’s Modulus (MPa)	Tensile Stress (MPa)	Strength at Break (N/Tex)	Energy at Break (J)
	Mean ± SD	Mean ± SD	Mean ± SD	Mean ± SD	Mean ± SD
***x*-Axis**					
1	331.93^abc^ ± 14.99	32.74^bcd^ ± 5.04	3.74^a^ ± 0.43	56.20^a^ ± 6.51	11.31^c^ ± 1.49
2	301.12^bc^ ± 14.58	33.01^bcd^ ± 2.22	3.41^ab^ ± 0.65	51.23^ab^ ± 6.84	10.07^c^ ± 1.25
3	264.53^cd^ ± 11.94	36.40^b^ ± 5.93	2.94^ab^ ± 0.46	44.18^bc^ ± 6.91	8.18^c^ ± 1.58
4	201.08^d^ ± 8.26	42.22^a^ ± 3.73	2.44^bc^ ± 0.94	36.74^c^ ± 4.20	6.74^c^ ± 0.77
5	338.46^abc^ ± 12.29	2.52^gh^ ± 0.64	1.34^de^ ± 0.27	20.10^d^ ± 4.07	3.25^c^ ± 0.94
6	325.44^bc^ ± 12.61	2.44^gh^ ± 0.98	1.27^de^ ± 0.21	19.17^de^ ± 4.79	2.92^c^ ± 1.02
7	305.33^bc^ ± 10.43	1.60^h^ ± 0.61	1.07^de^ ± 0.22	16.08^defg^ ± 3.34	2.22^c^ ±0. 63
8	298.44^bc^ ± 10.99	2.09^gh^ ± 0.78	1.13^de^ ± 0.31	17.09^def^ ± 4.72	2.42^c^ ±0. 80
***y*-Axis**					
1	311.12^bc^ ± 15.14	30.71^cde^ ± 1.41	3.18^ab^ ± 0.61	9.85^defg^ ± 1.99	47.71^a^ ± 3.17
2	215.44^d^ ± 15.48	26.49^e^ ± 2.45	1.82^cd^ ± 0.25	5.56^g^ ± 0.82	27.34^b^ ± 1.32
3	320.34^bc^ ± 12.62	29.04^de^ ± 2.34	3.15^ab^ ± 0.35	9.60^defg^ ± 0.96	47.34^a^ ± 4.17
4	323.88^bc^ ± 13.22	35.57^bc^ ± 2.54	3.78^a^ ± 0.84	11.93^defg^ ± 1.99	56.77^a^ ± 4.65
5	405.83^a^ ± 10.84	9.61^f^ ± 1.61	0.89^de^ ± 0.12	10.62^defg^ ± 0.98	2.21^c^ ± 0.26
6	302.26^bc^ ± 10.76	6.69^fg^ ± 0.92	0.42^e^ ± 0.08	6.25^fg^ ±0.33	1.50^c^ ± 0.14
7	331.87^abc^ ± 11.21	7.65^f^ ± 0.61	0.67^e^ ± 0.06	8.65^efg^ ±0.61	1.98^c^ ± 0.12
8	373.53^ab^ ± 11.89	8.38^f^ ± 0.91	0.72^e^ ± 0.07	9.25^defg^ ± 0.82	2.38^c^ ± 0.22

Superscript letters refer to a significant difference among different processed films.

**Table 2:** Differential scanning calorimetry (DSC) and thermogravimetric (TG) analysis of eight different specimens.

Composite Films	DSC		TG				
	Glass Transition (°C)	Crystallinity (%)	Onset Temperature (°C)	Inflection Temperature (°C)	End Temperature (°C)	Mass Change (%)	Residual Mass (%)
1	111.43	25.68	465.5	488.8	493.1	98.77	0.9
2	102.96	20.32	461.0	482.7	492.8	98.59	0.91
3	102.85	19.73	462.5	479.3	490.2	98.68	1.11
4	102.45	16.79	462.1	482.3	492.1	97.91	1.37
5	112.31	26.09	461.9	474.7	492.0	99.13	0.37
6	101.99	22.85	461.5	476.0	492.4	98.8	0.79
7	101.57	21.01	464.3	481.6	491.6	98.4	1.34
8	101.56	19.13	464.0	480.8	491.9	97.98	1.24

**Table 3:** Far-infrared emissivity, light transmittance, and moisture permeability of the composite films.

Composite Films	Far-Infrared Emissivity (ε)	Light Transmittance (%)	Moisture Permeability (g/m^2^·day)
1	0.512	14.7	0.27
2	0.861	13.1	0.32
3	0.892	12.4	0.4
4	0.921	10.9	0.45
5	0.508	71.3	1.73
6	0.866	67.9	2.17
7	0.89	67.6	3.27
8	0.924	57.6	3.38

## References

[1] Wang J.L., Lin Y.C., Young T.H., Chen M.H. (2019). Far-infrared ray radiation promotes neurite outgrowth of neuron-like PC12cells through AKT1 signaling. J. Formos. Med. Assoc..

[2] Sobajima M., Nozawa T., Ihori H., Shida T., Ohori T., Suzuki T., Matsuki A., Yasumura S., Inoue H. (2013). Repeated sauna therapy improves myocardial perfusion in patients with chronically occluded coronary artery-related ischemia. Int. J. Cardiol..

[3] Tao Y.F., Li T.H., Yang C.X., Wang N.X., Yan F., Li L. (2018). The Influence of Fiber Cross-Section on Fabric Far-Infrared Properties. Polymers.

[4] Hwang S., Lee D.H., Lee I.K., Park Y.M., Jo I. (2014). Far-infrared radiation inhibits proliferation, migration, and angiogenesis of human umbilical vein endothelial cells by suppressing secretory clusterin levels. Cancer Lett..

[5] Vatansever F., Hamblin M.R. (2012). Far infrared radiation (FIR): Its biological effects and medical applications. Photonics Lasers Med..

[6] Wang F., Liang J.S., Tang Q.G., Li L.W., Han L.J. (2010). Preparation and Far Infrared Emission Properties of Natural Sepiolite Nanofibers. J. Nanosci. Nanotechnol..

[7] Liang J.S., Zhu D.B., Meng J.P., Wang L.J., Li F.P., Liu Z.G., Ding Y., Liu L.H., Liang G.C. (2008). Performance and application of far infrared rays emitted from rare earth mineral composite materials. J. Nanosci. Nanotechnol..

[8] Chen G.W., Luo H.Y., Wu S.J., Guan J., Luo J., Zhao T.S. (2018). Flexural deformation and fracture behaviors of bamboo with gradient hierarchical fibrous structure and water content. Compos. Sci. Technol..

[9] Huang Z.H., Wang T., Zhang X.L., Zheng L.N., Xue G., Liang J.S. (2016). Preparation of tourmaline/graphene oxide and its application in thermal interface materials. J. Compos. Mater..

[10] Nikolic P.M., Paraskevopoulos K.M., Zachariadis G., Valasiadis O., Zorba T.T., Vujatovic S.S., Nikolic N., Aleksic O.S., Ivetic T., Cvetkovic O. (2012). Far infrared study of local impurity modes of Boron-doped PbTe. J. Mater. Sci..

[11] Deng Y., Zhang K.W., Yang Y.Y., Shi X.Y., Yang L., Yang W.Z., Wang Y., Chen Z.G. (2019). Ce/Mn dual-doped LaAlO_3_ ceramics with enhanced far-infrared emission capability synthesized via a facile microwave sintering method. J. Alloys Compd..

[12] Deng Y., Zhang K.W., Shi X.Y., Dong T.S., Yang L., Yang W.Z., Hong M., Wang Y., Dargusch M., Chen Z.G. (2019). Exploring the underlying mechanisms behind the increased far infrared radiation properties of perovskite-type Ce/Mn co-doped ceramics. Mater. Res. Bull..

[13] Caminero M.A., Chacon J.M., Garcia-Plaza E., Nunez P.J., Reverte J.M., Becar J.P. (2019). Additive Manufacturing of PLA-Based Composites Using Fused Filament Fabrication: Effect of Graphene Nanoplatelet Reinforcement on Mechanical Properties, Dimensional Accuracy and Texture. Polymers.

[14] Ferrel-Alvarez A.C., Dominguez-Crespo M.A., Torres-Huerta A.M., Cong H.B., Brachetti-Sibaja S.B., Lopez-Oyama A.B. (2017). Intensification of Electrochemical Performance of AA7075 Aluminum Alloys Using Rare Earth Functionalized Water-Based Polymer Coatings. Polymers.

[15] Sebastian M.T., Jantunen H. (2010). Polymer-Ceramic Composites of 0–3 Connectivity for Circuits in Electronics: A Review. Int. J. Appl. Ceram. Technol..

[16] Leung T.K., Chen C.H., Lai C.H., Lee C.M., Chen C.C., Yang J.C., Chen K.C., Chao J.S. (2012). Bone and Joint Protection Ability of Ceramic Material with Biological Effects. Chin. J. Physiol..

[17] Leung T.K., Lin Y.S., Lee C.M., Chen Y.C., Shang H.F., Hsiao S.Y., Chang H.T., Chao J.S. (2011). Direct and Indirect Effects of Ceramic Far Infrared Radiation on the Hydrogen Peroxide-scavenging Capacity and on Murine Macrophages under Oxidative Stress. J. Med. Biol. Eng..

[18] Jiang M.M., Zhang B., Tang X.N., Liu Q.L., Tian S.L. (2018). Preparation and characterization of hybrid antimicrobial materials based on Zn-Lu composites. J. Mater. Sci..

[19] Bhaskar R., Li J.L., Xu L.J. (1994). A Comparative-Study of Particle-Size Dependency of Ir and Xrd Methods for Quartz Analysis. Am. Ind. Hyg. Assoc. J..

[20] Heath C., Pejcic B., Delle Piane C., Esteban L. (2016). Development of far-infrared attenuated total reflectance spectroscopy for the mineralogical analysis of shales. Fuel.

[21] Greffrath F., Gorewoda J., Schiemann M., Scherer V. (2014). Influence of chemical composition and physical structure on normal radiant emittance characteristics of ash deposits. Fuel.

[22] Sabola K.D.A., Fechine P.B.A., Santos M.R.P., Freire F.N.A., Pereira F.M.M., Sombra A.S.B. (2007). Composite screen-printed thick films for high dielectric constant devices: Bi_4_Ti_3_O_12_-CaCu_3_Ti_4_O_12_ films. Polym. Compos..

[23] Xiong Y.B., Huang S.Y., Wang W.Q., Liu X.H., Li H.B. (2017). Properties and Applications of High Emissivity Composite Films Based on Far-Infrared Ceramic Powder. Materials.

[24] Kim S., Park J.C. (2010). Environment-friendly Hwangtoh Composite Materials Using Water Soluble Resin for Indoor Air Quality and Human Health. J. Compos. Mater..

[25] Defebvin J., Barrau S., Lyskawa J., Woisel P., Lefebvre J.M. (2017). Influence of nitrodopamine-functionalized barium titanate content on the piezoelectric response of poly(vinylidene fluoride) based polymer-ceramic composites. Compos. Sci. Technol..

[26] Ganfoud R., Puchot L., Fouquet T., Verge P. (2015). H-bonding supramolecular interactions driving the dispersion of kaolin into benzoxazine: A tool for the reinforcement of polybenzoxazines thermal and thermo-mechanical properties. Compos. Sci. Technol..

[27] Dayyoub T., Maksimkin A.V., Kaloshkin S., Kolesnikov E., Chukov D., Dyachkova T.P., Gutnik I. (2019). The Structure and Mechanical Properties of the UHMWPE Films Modified by the Mixture of Graphene Nanoplates with Polyaniline. Polymers.

[28] Qin C.R., Lu W., He Z.L., Qi G.S., Li J.L., Hu X.M. (2019). Effect of Silane Treatment on Mechanical Properties of Polyurethane/Mesoscopic Fly Ash Composites. Polymers.

[29] Soares A.R., Ponton P.I., Mancic L., d’Almeida J.R.M., Romao C.P., White M.A., Marinkovic B.A. (2014). Al_2_Mo_3_O_12_/polyethylene composites with reduced coefficient of thermal expansion. J. Mater. Sci..

[30] Ma L.C., Wu G.S., Zhao M., Li X.R., Han P., Song G.J. (2018). Modification of carbon fibers surfaces with polyetheramines: The role of interphase microstructure on adhesion properties of CF/epoxy composites. Polym. Compos..

[31] Ayyoob M., Kim Y.J. (2018). Effect of Chemical Composition Variant and Oxygen Plasma Treatments on the Wettability of PLGA Thin Films, Synthesized by Direct Copolycondensation. Polymers.

[32] Hu X.L., Tian M.W., Qu L.J., Zhu S.F., Han G.T. (2015). Multifunctional cotton fabrics with graphene/polyurethane coatings with far-infrared emission, electrical conductivity, and ultraviolet-blocking properties. Carbon.

